# Machine learning optimization of environmental factors influencing biomass and nutritional composition in local algal species

**DOI:** 10.1098/rsos.241336

**Published:** 2025-04-30

**Authors:** Aisha Khan, Saleem Ullah, Rifat Ali, Mahwish Rehman, Said Moshawih, Khang Wen Goh, Long Chiau Ming, Lai Ti Gew

**Affiliations:** ^1^ Department of Agricultural Chemistry and Biochemistry, The University of Agriculture, Peshawar, Pakistan; ^2^ Directorate of Soil and Plant Nutrition, Agriculture Research Institute, Tarnab, Peshawar, Pakistan; ^3^ Directorate General Agriculture Research, Government of Khyber Pakhtunkhwa , Peshawar, Pakistan; ^4^ Department of Pharmaceutical Sciences, Faculty of Pharmacy, Al-Ahliyya Amman University, Amman, Jordan; ^5^ Faculty of Data Science and Information Technology, INTI International University, Nilai, Malaysia; ^6^ Sir Jeffrey Cheah Sunway Medical School, Faculty of Medical and Life Sciences, Sunway University, Sunway City, Malaysia; ^7^ Datta Meghe College of Pharmacy, Datta Meghe Institute of Higher Education and Research (deemed to be University), Sawangi (M), Wardha India

**Keywords:** algae, biodiesel production, sustainable energy, machine learning, clean fuel, green product

## Abstract

Algae are recognized for their potential in biofuel production due to their high biomass yield, protein and lipid. This study investigates the influence of pH, temperature, light intensity, light colour and CO_2_ concentration on biomass and biochemical composition in five algal genera (*Chlorella, Botryococcus, Chlamydomonas, Tetraselmis* and *Closterium*). Algal samples were isolated from aquatic environments in KPK-Pakistan and cultured under controlled conditions. Environmental variables were systematically varied: pH, temperature, light intensity, light colour and CO_2_ concentration. Biochemical analyses revealed biomass ranging from 0.2 to 2.1 g l^−1^, lipids 7.2–24.5% and proteins 8–49.5%, with optimal conditions of pH 7, 30°C, red light, 3000 lux and 9% CO₂. Machine learning was applied to optimize environmental conditions, with random forest (RF) identified as the most effective model. A novel metric, *W*_new, combining performance and error metrics, facilitated robust model evaluation and hyperparameter tuning. The model’s feature importance analysis ranked CO₂ concentration and pH as the most influential factors. RF achieved *R*² scores of 0.686 (training) and 0.534 (validation), demonstrating strong predictive performance. This study integrates experimental and computational approaches, providing a detailed framework for optimizing algal cultivation. We highlighted the utility of machine learning in enhancing biomass and lipid productivity, advancing the sustainable production of biofuel.

## 1. Introduction

Given the depletion of fossil fuel reserves and the limitations of first-generation biofuels, optimizing biomass production for biofuel is essential. Algae, which inhabit diverse terrestrial and aquatic environments, have attracted significant attention in biofuel technology due to their high oil content, adaptability and potential for cultivation in photobioreactors [1]. Notably, certain algal species can produce biomass with oil content exceeding 50%, positioning algal biofuel as a promising alternative energy source [2]. Microalgae, in particular, are recognized for their high water and oil contents, making them economically viable for energy production. These organisms can thrive in suboptimal water conditions and confined spaces, rapidly completing their life cycle with appropriate nutrients and aeration, thus requiring significantly less land compared with traditional oil crops [3].

The growth of microalgae is influenced by a variety of biotic and abiotic factors, with the latter, including light, CO_2_, water, minerals, temperature and pH, playing crucial roles. Temperature, for instance, profoundly affects the physiological and morphological responses of algae. Studies have shown that lower temperatures can hinder metabolic processes, while higher temperatures accelerate growth. Optimal temperatures vary among genera, with *Isochrysis* thriving at 24–26°C and *Tetraselmis* and *Nannochloropsis* at 19–21°C, generally achieving higher biomass at approximately 23°C [4,5]. Effective temperature control is thus essential for outdoor cultivation.

pH levels significantly influence nutrient uptake, biochemical reactions, metabolic processes and the bioavailability of CO_2_ for photosynthesis in algae. The optimal pH range for algal growth typically lies between 6 and 8. Variations in daily pH can alter nutrient availability and photosynthetic efficiency, consequently affecting biomass production. Elevated pH levels can induce physiological stress and reduce the availability of essential macronutrients, thereby impacting overall algal growth and productivity [6,7]. Additionally, microalgae require a suite of macronutrients, including carbon, nitrogen, phosphorus, sulfur, calcium and magnesium, as well as trace elements like iron, boron, manganese and copper. These nutrients can be sourced from minerals or supplied through bacterial metabolism. Certain species utilize carbonates such as Na_2_CO_3_ and NaHCO_3_ for growth, with carbonic anhydrase activity aiding CO_2_ assimilation and influencing medium pH [8,9]. Direct bicarbonate uptake is also observed in some species, facilitating growth and biomass accumulation [10].

Machine learning (ML) models have emerged as powerful tools in optimizing environmental factors to maximize biomass production [11]. These models can analyse complex datasets to identify critical variables and predict optimal conditions. For instance, a study by Camacho-Rodríguez *et al*. [12] utilized genetic algorithms to optimize the culture medium for *Nannochloropsis gaditana*, resulting in a 23% increase in eicosapentaenoic acid (EPA) productivity. Similarly, another study by Sultana *et al*. [13] developed nonlinear empirical models using support vector regression (SVR) to investigate the effects of light–dark cycles and nitrogen (NaNO_3_) doses on the growth rate, biomass productivity and lipid productivity of *Chlorella kessleri*. The SVR models, optimized with a Bayesian algorithm and combined with a crow search algorithm, demonstrated superior performance over response surface methodology models and identified the optimal conditions for maximizing these parameters. These examples underscore the potential of integrating ML with traditional experimental approaches to enhance the efficiency and productivity of algal cultivation. In the same context, a study by Nayak *et al*. [14] employed artificial neural networks to optimize for cleaner production of biomass with co-valorization of wastewater and flue gas in an algal biorefinery. Similarly, another study by Zarkami *et al*. [15] utilized decision tree model to predict the abundance of *Dunaliella salina* in the Meighan wetland, revealing that environmental factors such as sunny hours, dissolved oxygen and sodium concentrations significantly influence its habitat suitability, while total suspended solids and precipitation negatively impact its abundance.

We hypothesize that environmental factors such as pH, temperature, light intensity, light colour and CO₂ concentration significantly influence the biomass yield and biochemical composition (e.g. lipid and protein content) of algae, with certain conditions leading to optimized outcomes for biofuel production. This study aims to optimize the production of biomass and lipid content for biofuel applications while maintaining adequate levels of other biochemical properties, such as protein and ash, to ensure the versatility of algal products. By systematically examining the impact of these factors, we seek to identify high-oil-content species suitable for potential biodiesel production. The experimental design employs a factorial approach to independently assess the influence of each environmental parameter, thereby simplifying the analysis of their effects on algal physiological responses. Advanced statistical methods, including multivariate analysis of variance (MANOVA), are utilized to evaluate the collective impact of these factors on multiple dependent variables simultaneously. Furthermore, feature selection using ML models provides insights into the most influential environmental conditions for optimizing biomass and nutrient composition.

## 2. Material and methods

**Figure 1.**
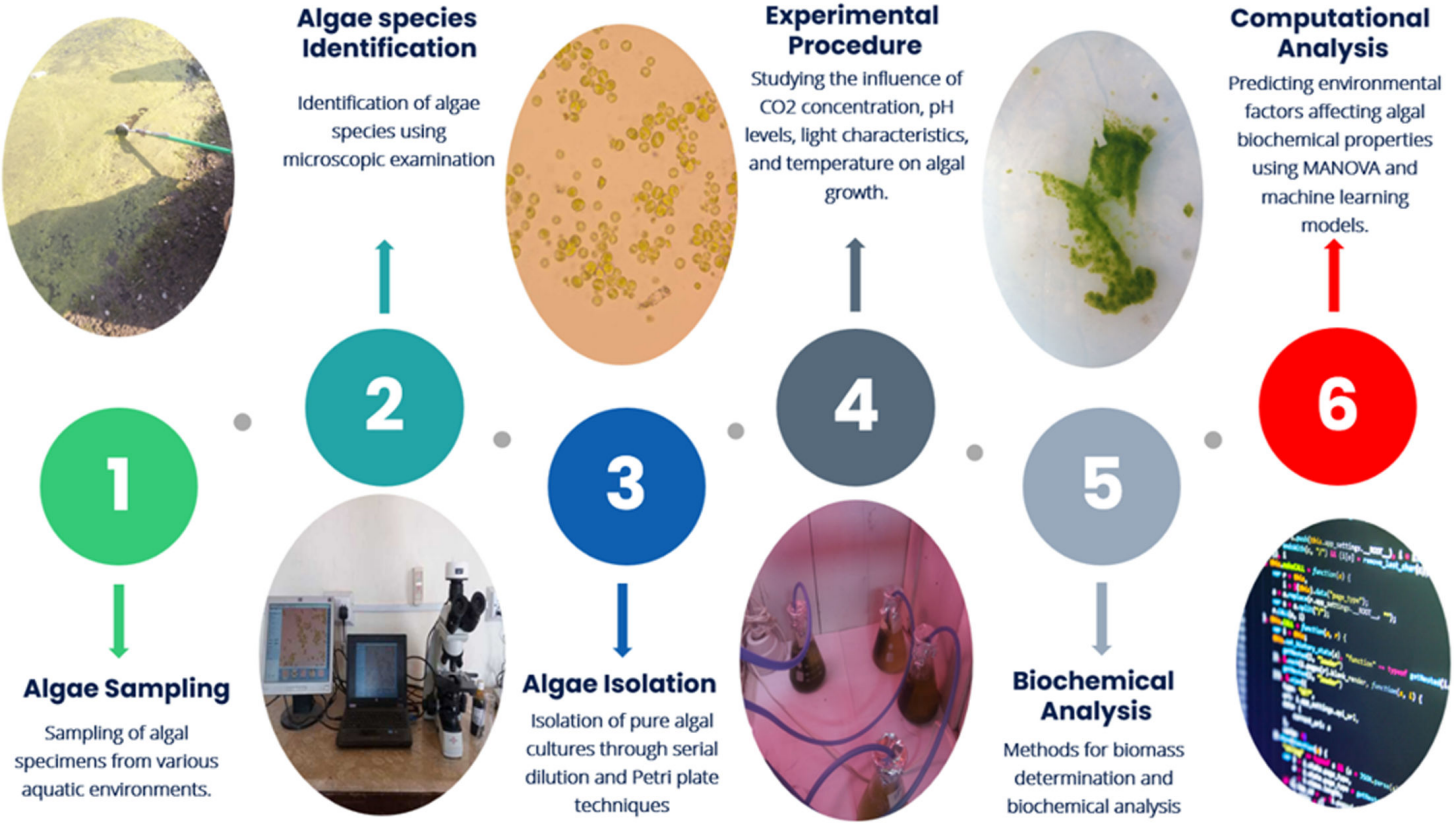
Schematic of the experimental and computational procedures for algal study.

The experimental procedures for this study were meticulously designed to investigate the growth and biochemical composition of algal species under varying environmental conditions. As illustrated in figure 1, the methods encompass a comprehensive workflow, beginning with the sampling of algal specimens from diverse aquatic environments in Pakistan, followed by their identification, isolation and cultivation. The influence of environmental factors on algal growth was systematically studied. Subsequently, detailed biochemical analyses were conducted to quantify biomass and various biochemical constituents. Finally, advanced computational techniques were employed to analyse the data and predict key environmental factors affecting algal properties.

### 2.1. Experimental procedure

#### 2.1.1. Sampling

Algal specimens were harvested from a variety of aquatic environments, including ponds, lakes and still waters, spanning the regions of Peshawar, Charsadda, Nowshera, Mardan, Swabi, Dargai and Swat. These specimens were collected using medium-sized transparent plastic containers, to which purified water was added at a volume twice that of the algal mass. Subsequently, these specimens were transported to the Biofuel Laboratory with due diligence, where they were rinsed with distilled water. Following this, the specimens were relocated into glass bottles with a capacity of 1 l. Within these containers, Bold’s basal medium (BBM) [16] was introduced to facilitate the growth of the algae.

#### 2.1.2. Identification of algae

Microscopic slides of the collected algal samples were prepared in accordance with the methodology outlined by Manoylov [17]. These prepared slides were subsequently examined using a Nikon compound light microscope equipped with a digital camera attachment, which was connected to a computer for imaging and documentation. This set-up allowed for high-resolution visualization and facilitated precise morphological observations. Through this examination process, and with the assistance of the identification manual by Prescott [18], five distinct species were identified: *Chlorella* spp. [19], *Botryococcus* spp. [20], *Chlamydomonas* spp. [21,22], *Tetraselmis* spp. [23] and *Closterium* spp. [24]. The morphological characteristics of these species, as observed in this study, were found to be identical to those described in the referenced publications. This confirmation further supports the accuracy of the identification process. These species were selected for further investigation based on their relevance to the study’s objectives and the clarity and distinctiveness of their observed morphological features under the microscope.

#### 2.1.3. Isolation

The isolation of the identified algal samples was conducted utilizing serial dilution and Petri plate culture techniques. Initially, 100 ml of BBM and 5 ml of the algal sample were combined in a 250 ml Erlenmeyer flask. Subsequently, 5 ml of this mixture was transferred to another flask and diluted to 100 ml with the same medium, a procedure that was repeated three to four times. These steps were performed in triplicate to ensure the consistency of the results. These flasks were then exposed to sunlight for a period of 3–5 days, after which algal growth was observed visually. The selected species were subsequently cultured in test tubes over a span of 7 days. The growth observed on the seventh day was meticulously analysed through microscopic examination for further identification.

For the purpose of further isolation, cultures from the flasks were subcultured onto Petri plates. This process involved transferring a single drop of the culture from the flask onto a Petri plate using a micropipette and spreading it in streaks to facilitate isolation. The Petri dishes were labelled and placed under sunlight to promote growth.

By the fifth day, distinct colonies were individually segregated using forceps and re-cultured on new Petri plates for an additional 4 days. Following this period, the cultures were examined under a light microscope. In instances of contamination, the cultures were subjected to repeated re-culturing on Petri plates to achieve a pure culture. Once a pure culture was established, it was transferred to a 1 l media bottle for preservation and future experimental applications. This systematic approach ensured the isolation of pure algal cultures for detailed study and examination.

#### 2.1.4. Influence of CO_2_ concentration, pH levels, light characteristics and temperature on the growth and biochemical composition of algal species

This study investigated the impact of various environmental parameters, including CO_2_ concentration, pH levels, light colour and intensity, and temperature, on the growth and biochemical composition of algal species. Algae were cultured in BBM medium with four CO_2_ concentrations (5, 7, 9 and 11%) using air pumps for consistent CO_2_ delivery [25]. Additionally, pH levels were adjusted to 5, 7, 9 and 11 using 1 N NaOH and 1 N hydrochloric acid (HCl) [26] and were monitored and adjusted daily with a pH meter. Four different light sources—white, red, green and blue—were selected for optimization [27], and light intensities of 2000, 2500, 3000 and 3500 lux were studied. Additionally, algal species were exposed to thermal treatments at 10, 20, 30 and 40°C. All experiments were conducted in triplicate to ensure the reliability of the results.

The incubation period lasted for 7 days, after which the biomass was harvested and quantified. Biochemical analyses were performed to determine the contents of crude lipids, crude proteins, fibre, nitrogen-free extract (NFE), sodium (Na), potassium (K), ash and moisture. These analyses were crucial for understanding the effects of different CO_2_ levels on the growth and biochemical composition of the algal species, thereby providing insights into their potential applications in biofuel production and other biotechnological processes.

#### 2.1.5. Biomass and other biochemical constituents

##### 2.1.5.1. Biomass

Biomass was determined by the gravimetric method [28]. Samples of growing algae were taken at 7 day intervals. The biomass was filtered using pre-weighed filter paper. After oven drying at a moderate temperature, the weight was recorded as biomass per portion, subtracting the mass of the filter paper. The collected biomass was centrifuged for 10 min at 8000 r.p.m. on the seventh day. The pellet was collected and oven dried at 60°C. Biomass was then determined gravimetrically and expressed in g l^−1^ [26]. All measurements were conducted in triplicate to ensure accuracy and reproducibility.

##### 2.1.5.2. Lipids

The per cent crude fat content was quantified using the Soxhlet extraction method as described by Paez *et al.* [29]. A dried algae sample, approximately 2 g in mass, was enveloped in filter paper and placed inside a thimble within the extraction apparatus. A clean beaker was attached to the system and filled to one third of its capacity with n-hexane. The extraction process was carried out until the n-hexane had completely evaporated. The per cent crude fat was then calculated using the following formula. All lipid analyses were performed in triplicate to ensure consistency and reliability.


$$Fat\;content\;\%=\frac{\left(net\;ether\;extract\;weight\right)}{Sample\;weight}*100\%$$

##### 2.1.5.3. Mineral analysis

Mineral analysis was carried out using the flame spectrophotometry method as outlined by Paez *et al.* [29]. This technique involves the use of a flame photometer to measure the concentration of various minerals in the sample. The sample is atomized in a flame, and the intensity of the emitted light at characteristic wavelengths is measured, which corresponds to the concentration of specific minerals such as Na and K. All mineral analyses were performed in triplicate to ensure accuracy and reproducibility.

##### 2.1.5.4. Proximate analysis

The proximate composition, including moisture, ash, crude protein, crude fat, fibre and NFE, was determined using standard AOAC methods [29]. The analyses were performed on a dry matter basis and in triplicate to ensure accuracy and reproducibility.

##### 2.1.5.5. Moisture content

Determined by the oven drying method, a 1 g sample was placed in pre-weighed Petri dishes and dried in an oven at 105°C for 5−6 h until a constant weight was achieved. The moisture content was calculated based on the weight loss during drying. Per cent moisture content was obtained by the following formula. Moisture-free samples were kept in clean and airtight bottles which were then used for further analysis. All moisture content determinations were performed in triplicate to enhance reliability and precision.


$$Moisture\;\%\;=\frac{W1-W2}{weight\;of\;sample}\times 100,$$

where *W*1 is the initial weight of the sample and *W*2 is the final weight of the sample.

##### 2.1.5.6. Ash content

Determined using a muffle furnace. The dried sample was placed in a crucible, charred using a burner and then ashed at 550°C for 5−6 h until a greyish residue was obtained. The ash content was calculated based on the weight of the residue relative to the initial sample weight. Ash content was calculated by the following formula:


$$Ash\%\;=\;\frac{W3-W1}{weight\;of\;sample}\times 100,$$

where *W*1 is the weight of the empty crucible (before adding the sample) and *W*3 is the weight of the crucible plus the ash residue after the combustion process in the muffle furnace.

##### 2.1.5.7. Crude fibre determination

The acid–base digestion method was employed to assess the crude fibre content as a percentage. Initially, 2 g of the sample was placed in a beaker containing 100 ml of 0.1 M HCl. A condenser was affixed to the beaker, which was then heated for 5 min, followed by uniform boiling for approximately 30 min. Subsequently, the solution was allowed to cool, after which 100 ml of NaOH was added and boiled for an additional 35 min. Just before the end of the boiling period, 0.5 g of Na_2_EDTA was introduced. The solution was then transferred to a crucible attached to a filtration apparatus. The filtrated sample was dried in a crucible in an oven at 100–130°C for 2 h or overnight. After drying, the sample was cooled and weighed again. Ash formation was conducted using a muffle furnace, where the sample was subjected to a temperature of 550°C for 6 h. After cooling, the weight was recorded once more. All analyses were performed in triplicate to ensure accuracy and reproducibility. The percentage of crude fibre (CF) in the sample was calculated using the following formula:


$$\%CF\;in\;sample=\frac{\left(weight\;of\;crucible+weight\;of\;dry\;crucible\right)-\left(weight\;of\;crucible+ash\;residue\right)}{weight\;of\;crucible+sample-weight\;of\;empty\;crucible}*100.$$

##### 2.1.5.8. Crude protein determination

Crude protein content was assessed using the Kjeldahl method, a standard technique in analytical chemistry. Initially, the sample underwent digestion by heating it with concentrated sulfuric acid (H_2_SO_4_). During this process, hydrolysis occurred in the presence of a digestion mixture containing potassium sulfate (K_2_SO_4_) and copper sulfate (CuSO_4_) in an 8 : 1 ratio. The resulting acidic solution was subsequently rendered alkaline by the addition of sodium hydroxide (NaOH). Ammonium hydroxide (NH_4_OH) was formed, releasing ammonia (NH_3_), which reacted with boric acid (H_3_BO_3_) to produce ammonium borate ((NH_4_)_2_B_4_O_7_). This solution was then titrated against 0.05 N HCl. The amount of nitrogen (N) present in the sample was calculated from the titration volume. Finally, the nitrogen content was multiplied by a conversion factor of 6.25 to obtain the protein content. All analyses were performed in triplicate to ensure accuracy and reproducibility. This method provides a reliable determination of crude protein content in the sample.

##### 2.1.5.9. Nitrogen-free extract

NFE was computed as the difference, representing the carbohydrate content of the sample. The NFE value is derived by subtracting the percentages of crude protein (%CP), crude fibre (%CF), ash (%Ash) and crude fat (%C Fibre) from 100. This calculation provides an estimation of the carbohydrate fraction in the sample, excluding nitrogenous compounds, fibre, ash and fat.

### 2.1.6. Mineral contents

#### 2.1.6.1. Preparation of acid digest

The acid digest for mineral content analysis was prepared according to the AOAC [29] protocol. A 1 g sample was taken, and 10 ml of nitric acid (HNO_3_) was added, then left to react overnight. On the following day, 4 ml of perchloric acid (HClO_4_) was added, and the solution was heated until the perchloric acid evaporated as dense fumes. After the fumes dissipated, the digest was transferred to a 100 ml volumetric flask and diluted to 100 ml with distilled water. This prepared solution was then used for the analysis of Na and K. All digestion procedures were performed in triplicate to ensure consistency.

#### 2.1.6.2. Determination of Na and K

Sodium and potassium concentrations were determined using a flame photometer Sherwood 410, UK. The photometer was calibrated with Na and K standards ranging from 0 to 100 μg g^−1^. For sodium measurements, the photometer was set to the sodium filter at 589 nm, and for potassium measurements, it was set to the potassium filter at 769 nm. Emission readings were recorded, and a standard curve was generated from these standard solutions.

A 5 ml aliquot of the acid digest was placed into a container, and the emissions of Na and K were measured using a capillary tube in the photometer. The concentrations of Na and K in the sample were then computed from the standard curve using the following formula. All measurements were performed in triplicate to ensure accuracy and reproducibility.


$$mg/100\;g\;of\;sodium\;and\;potassium=\left(graphical\;value\times d.f.\times \frac{100}{weight\;of\;sample}\right).$$

### 2.2. Computational procedure

#### 2.2.1. Multivariate analysis for environmental factors impact on algal biochemical properties

Following data preprocessing across algal genera (*Closterium, Tetraselmis, Chlamydomonas, Botryococcus* and *Chlorella*), a MANOVA was performed to simultaneously assess the impact of experimental factors on multiple response variables related to growth and composition. This statistical technique extends ANOVA to multiple dependent variables, allowing for the simultaneous assessment of predictor variables on a set of response variables. The dataset included several biochemical properties as dependent variables, with the factor name as the independent variable. The MANOVA model was specified to incorporate these variables, and the analysis provided various multivariate test statistics, including Wilks’ lambda, Pillai’s trace, Hotelling–Lawley trace and Roy’s greatest root. These statistics measure the variance in the dependent variables explained by the independent variable(s) and provide a comprehensive evaluation of the significance and extent of these effects. Degrees of freedom, *F*-values, and *p*-values were calculated for each test statistic, indicating the statistical significance of the results. This approach allowed us to assess the collective impact of environmental factors on the biochemical properties of algae, providing valuable insights into the relationships within the dataset. Additionally, a MANOVA was conducted to evaluate the overall impact of the factors on multiple dependent variables simultaneously. All calculations, ML modelling and figure visualization were performed using Python and Jupyter Lab 3.6.3.

#### 2.2.2. Model selection for identifying key environmental factors affecting algal biochemical properties

To determine the environmental factors with the highest impact on the biochemical properties of algae, such as biomass, protein, lipid, fibre, ash, Na, K, moisture and NFE, we employed a comprehensive ML approach developed by Moshawih *et al*. [30]. Bayesian optimization, while a powerful method for hyperparameter tuning in scenarios with expensive function evaluations [31], has inherent limitations. It builds a probabilistic model of the objective function to identify hyperparameters that maximize an acquisition function [32]. However, this process can become computationally expensive, particularly in large parameter spaces or when models are complex. Each iteration of Bayesian optimization involves updating the probabilistic model and retraining on the dataset, significantly increasing computational overhead as the number of evaluations grows [33].

In contrast, the method we employed integrates multiple performance metrics through W_new, offering distinct advantages in balancing trade-offs between bias and variance. By concurrently evaluating predefined metrics, W_new streamlines the optimization process, reducing the need for repetitive model training and thus improving computational efficiency. This approach is particularly beneficial for datasets with complex environmental interactions, where rapid and robust model evaluation is critical.

Additionally, SHAP-based feature selection provides an alternative with unique strengths in feature attribution. Rooted in game theory, SHAP offers detailed global and local explanations of feature contributions, enabling comprehensive insights into model behaviour. Its model-agnostic nature makes it adaptable across diverse ML models, ensuring consistency and interpretability. However, the computational demands of SHAP can be significant, especially when applied to large datasets or models with numerous features. SHAP’s evaluation of feature contributions across all possible coalitions introduces computational complexity, which may limit its practicality in certain scenarios [34].

Moreover, the technique for order of preference by similarity to ideal solution (TOPSIS) provides a structured framework for multi-criteria decision-making. By identifying ideal and anti-ideal solutions, TOPSIS evaluates alternatives based on their relative proximity to these benchmarks [35]. This approach facilitates the ranking of environmental factors, allowing for a comprehensive comparison across multiple criteria [36]. However, its effectiveness depends on accurately determining criteria weights and selecting reference alternatives, which can introduce subjectivity. Furthermore, the scalability of TOPSIS diminishes as the number of criteria and alternatives increases, adding to its computational burden in large datasets [36].

In contrast, the W_new framework minimizes computational overhead by predefining evaluation metrics that are calculated concurrently. This optimization eliminates repetitive model evaluations, enhancing the efficiency and robustness of the process. By integrating multiple metrics into a single framework, W_new is especially advantageous in scenarios requiring real-time model assessment or the evaluation of multiple models simultaneously, such as in algae cultivation optimization. This efficiency, combined with the ability to balance accuracy and error measures, makes it a practical and reliable solution for addressing complex environmental datasets. In contrast, the method using W_new optimizes the integration process by reducing the number of model evaluations needed. By predefining a set of evaluation metrics that are calculated concurrently, W_new minimizes the need for repetitive model training and thus offers a more computationally efficient approach. This efficiency is especially advantageous in scenarios where rapid model assessment is crucial, such as in real-time systems or when multiple models need to be evaluated simultaneously.

#### 2.2.3. Data preparation

Our goal was to identify the most predictive models for each biochemical property and understand the significance of various environmental influences. The data preparation phase involved preprocessing the dataset by converting categorical variables using one-hot encoding and excluding metadata columns. The dataset was split into 70% for training and 30% for testing, following standard practices in ML. We defined our features as all columns except for the target variables, which were the biochemical properties of interest. In addition, we employed StandardScaler to normalize the dataset before performing principal component analysis (PCA), ensuring that features with varying scales, such as pH, CO₂ concentration and light intensity, contributed equally to the principal components. This preprocessing step is critical in PCA to avoid dominance by features with larger numerical ranges.

#### 2.2.4. Model development and evaluation

We implemented a robust ML framework to optimize predictive models for algal biochemical properties. By conducting a thorough evaluation of 12 distinct ML models, we ensured the selection of the most accurate model for predicting these properties. The models assessed include KNeighborsRegressor, ElasticNet, LinearRegression, SVR, NuSVR, DecisionTreeRegressor, GradientBoostingRegressor, RandomForestRegressor and AdaBoostRegressor. To optimize model performance, we employed GridSearchCV for hyperparameter tuning, systematically exploring parameter ranges to identify optimal configurations. For instance, in the case of the RandomForestRegressor, the number of estimators was varied between 50 and 300, while the maximum depth ranged from 10 to 50. These parameter ranges are adjustable based on the specific requirements and characteristics of the model.

To address the high dimensionality of the feature space, we explored two dimensionality reduction approaches: PCA and feature selection methods. The selection of the most effective technique was guided by its ability to produce the most robust and accurate predictive model. The number of principal components in PCA was systematically varied between 5 and 15 to identify the optimal range for balancing explained variance and model complexity. The PCA analysis, conducted after scaling with StandardScaler, retained the first 10 components that explained over 85% of the variance in the data. This dimensionality reduction significantly improved model performance by reducing noise and computational complexity. The choice between PCA and feature selection, along with the optimal number of components or features, was rigorously evaluated using the GridSearchCV process. This integrated approach ensured that the dimensionality reduction strategy was tailored to maximize the performance of each predictive model.

**Figure 2.**
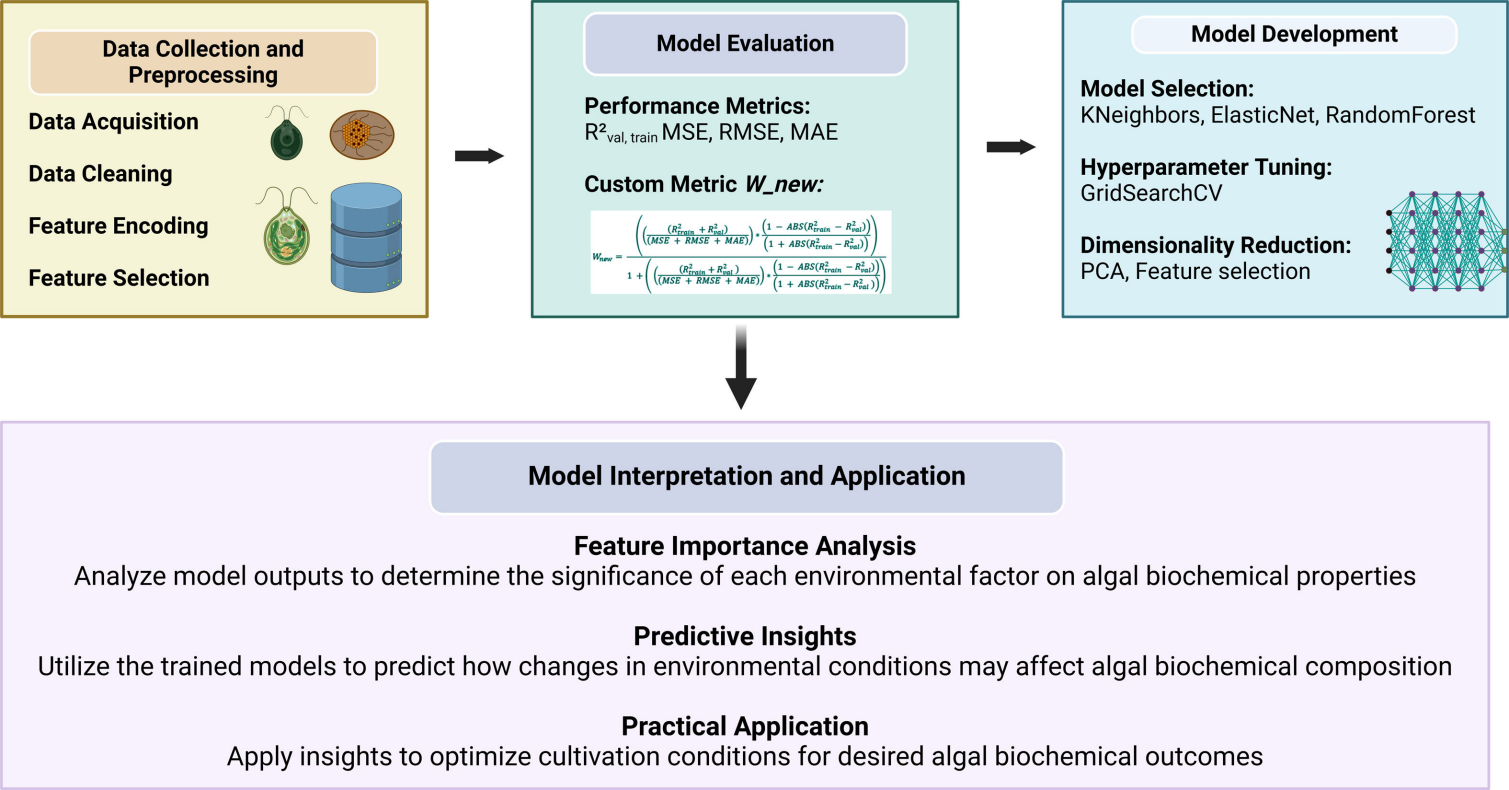
Framework for machine learning-based prediction and optimization of algal biochemical properties, from data collection to model application.


Model performance was primarily evaluated using *R*² scores. To further refine model selection and address trade-offs between overfitting and underfitting, the framework introduced a custom metric, W_new. This metric balances training and validation *R*² scores, mean squared error (m.s.e.), root mean squared error (RMSE) and mean absolute error (MAE), providing a more robust assessment of model performance. The framework utilized in this study is accessible at the GitHub Repository [37] and is designed for reproducibility and flexibility, allowing researchers to adapt it to different datasets and research contexts. By leveraging this framework, we achieved a nuanced evaluation of model performance, leading to more accurate predictions of algal biochemical properties as illustrated in figure 2.

$$W_{new}=\;\frac{\left(\left(\frac{\left(R_{train}^{2}+R_{val}^{2}\right)}{\left(MSE+RMSE+MAE\right)}\right)*\frac{\left(1-ABS\left(R_{train}^{2}\;-\;R_{val}^{2}\right)\right)}{\left(1+ABS\left(R_{train}^{2}-R_{val}^{2}\right)\right)}\right)}{1\;+\left(\;\left(\frac{\left(R_{train}^{2}+R_{val}^{2}\;\right)}{\left(MSE+RMSE+MAE\right)}\right)*\frac{\left(1-ABS\left(R_{train}^{2}\;-R_{val}^{2}\right)\right)}{\left(1+ABS\left(R_{train}^{2}-R_{val}^{2}\;\right)\right)}\right)}.$$

## 3. Results and discussion

This study investigates the impact of five environmental treatments: temperature, pH, CO_2_ concentration, light intensity and the light colour on nine biochemical parameters: biomass, fibre, lipid, protein, NFE, moisture, ash, sodium and potassium, as illustrated in electronic supplementary material, part 1. The five algae genera analysed were *Chlorella*, *Botryococcus*, *Chlamydomonas*, *Tetraselmis* and *Closterium* spp. By systematically varying these environmental factors, we aimed to identify optimal growth conditions and their effects on the biochemical composition of these algae species. The results provide insights into how each treatment influences the specified outcomes, contributing to the optimization of algal cultivation for nutritional and industrial applications.

### 3.1. Multivariate analysis and feature importance across algal species

The MANOVA results indicated that the factors significantly influenced the dependent variables across all algal species. The intercept tests confirmed the overall model’s significance, with Wilks’ lambda, Pillai’s trace, Hotelling–Lawley trace and Roy’s greatest root all showing highly significant results (Pr > *F* = 0.0000). Detailed results can be found in electronic supplementary material, part 2.

In this analysis, we aimed to identify the most suitable ML models for predicting the impact of various environmental factors (such as pH, temperature, CO_2_ and others) on maximizing or minimizing the biochemical properties of different algal species. By evaluating a range of models, we assessed their performance in predicting the most effective environmental conditions for properties such as biomass, protein, lipid, fibre, Ash, Na, K, moisture and NFE. The suitability of each model was determined using the custom metric *W_*new, which balances accuracy, consistency and error measures. In the subsequent stages, one selected model was employed to rank the importance of various environmental factors, providing insights into the key determinants influencing each biochemical property across different algal species, as illustrated in table 1.

**Table 1.** Performance metrics comparison of various machine learning models to select the best model for calculating environmental factors importance affecting the biochemical content in local microalgae.

metric	elastic regression	XGBoost	decision tree	KNN	NuSVR	linear regression	random forest	SVR	AdaBoost
*R*^2^ training score	0.564	0.404	0.532	0.551	0.466	0.716	0.686	0.391	0.373
*R*^2^ validation score	0.254	0.181	0.185	0.155	0.020	0.550	0.534	0.118	0.381
MAE	0.162	0.192	0.200	0.185	0.215	0.115	0.104	0.201	0.223
RMSE	0.233	0.244	0.244	0.248	0.267	0.217	0.145	0.254	0.245
m.s.e.	0.054	0.059	0.059	0.061	0.100	0.059	0.071	0.064	0.100
*W_*new	0.489	0.428	0.408	0.381	0.242	0.697	0.736	0.358	0.566

Table 1 presents a comprehensive comparison of performance metrics for various ML models used to identify the environmental factors that influence the biochemical composition of local microalgae. The models analysed include 12 well-known ML algorithms, evaluated using metrics such as *R*² (training and validation), MAE, RMSE, m.s.e. and a newly introduced weighted metric, *W*_new, which balances predictive accuracy and error rates for a more comprehensive assessment. Among these models, the random forest (RF) model demonstrated superior performance, achieving the highest *W*_new value of 0.736, indicating its robust reliability for modelling environmental factors. Its *R*² training and validation scores were 0.686 and 0.534, respectively, showcasing its ability to generalize well to unseen data. Furthermore, the RF model achieved the lowest MAE (0.104) and RMSE (0.145), underscoring its high accuracy across datasets.

This selection is supported by its consistent performance and extensive use in similar ecological studies [33]. For instance, Li *et al*. successfully employed the RF model to predict chlorophyll-a concentrations in a shallow lake, demonstrating its effectiveness in handling complex environmental datasets [38]. Its capacity to capture nonlinear relationships and interactions among variables further emphasizes its suitability for ecological studies. Based on its superior performance across all evaluated metrics, the RF model was chosen for our analysis to calculate the contribution of environmental factors to the biochemical content of local microalgae. This decision reflects its strong predictive capabilities, reliability and proven effectiveness in similar applications.

### 3.2. Random forest regression robustness, predictability and hyperparameter optimization

The performance of the RF regression model was comprehensively assessed in terms of robustness, predictability and hyperparameter optimization. Robustness was evaluated using k-fold cross-validation (CV), which was widely recognized for ensuring model reliability across different subsets of data. Various fold configurations were tested, and fivefold CV was identified as the optimal setting for the model, balancing computational efficiency with consistent performance. The *W*_new metric, a composite evaluation criterion combining *R*² (training and validation), MAE, RMSE and m.s.e., was employed to further assess robustness. This metric provided a holistic measure of the model’s robustness by balancing predictive accuracy with error minimization. The RF model exhibited minimal variance in error metrics across CV folds, highlighting its stability and reliability.

Predictability was assessed using standard regression evaluation metrics, which confirmed that the model generalized effectively to unseen data. The RF regression model achieved an *R*² score of 0.686 on the training dataset and 0.534 on the validation dataset, demonstrating strong generalization capabilities. Residual analysis was also performed, confirming unbiased predictions as the residuals displayed a normal distribution with a mean close to zero. Additionally, feature importance analysis was conducted to validate the model’s predictive capabilities, identifying key environmental factors, such as pH and CO₂ concentration, that significantly influenced algal biochemical properties. These findings aligned with known biological relationships, underscoring the model’s reliability in capturing meaningful patterns within the data.

Hyperparameter optimization was carried out using a systematic grid search approach combined with fivefold CV. Several critical hyperparameters were tested to enhance the model’s predictive accuracy and computational efficiency. The number of trees in the forest (n_estimators) was varied across several values, with 100 emerging as the optimal setting due to its balance between stability and computational cost. Similarly, the maximum tree depth (max_depth) was tested with multiple values, and a depth of 10 was selected as it provided sufficient flexibility to capture complex relationships while avoiding overfitting. For the minimum samples required to split a node (min_samples_split), several values were tested, and 5 yielded the best results by balancing granularity and generalization. Finally, the minimum samples required in a leaf node (min_samples_leaf) were tuned, and 5 was found to prevent overfitting by limiting the creation of small leaf nodes. The selected hyperparameters—n_estimators = 100, max_depth = 10, min_samples_split = 5, and min_samples_leaf = 5—offered the best combination of predictive accuracy and computational efficiency, aligning with established best practices for RF regression.

### 3.3. Feature importance ranking across environmental factors for biochemical optimization

The feature importance ranking was derived using the RF regression model, selected for its superior performance across multiple evaluation metrics, including *R*², MAE, RMSE and the custom metric *W*_new. RFs inherently provide a measure of feature importance by evaluating the decrease in impurity, such as Gini impurity or entropy, each time a feature is used to split the data. The more a feature reduces impurity, the more important it is considered [39,40].

This method, known as mean decrease in impurity, sums the reduction in impurity brought by a feature across all trees in the forest. The steps include:

(1) Training multiple decision trees on various sub-samples of the dataset.
(2) For each tree, measuring the reduction in impurity (e.g. Gini impurity) brought by each feature at each split.
(3) Averaging these reductions across all trees to obtain the final importance score for each feature [41].

This approach is widely recognized and utilized in ML for assessing feature importance. For instance, the scikit-learn library provides an implementation of this method, computing feature importances as the mean and standard deviation of the accumulation of impurity decrease within each tree [42]. By applying this methodology, we identified the relative contributions of environmental factors, such as CO₂ concentration, pH and light intensity, to biochemical properties including biomass, protein and lipid content. This process is detailed in §2 (see table 1 for model comparison and performance metrics), and its robustness was validated using CV and statistical tests (MANOVA results). Further details on the calculation methodology and ranking outcomes are provided in electronic supplementary material, part 3.

### 3.4. The effect of environmental factors on biomass and other biochemical and nutritional compositions of *Chlorella* spp.

**Figure 3.**
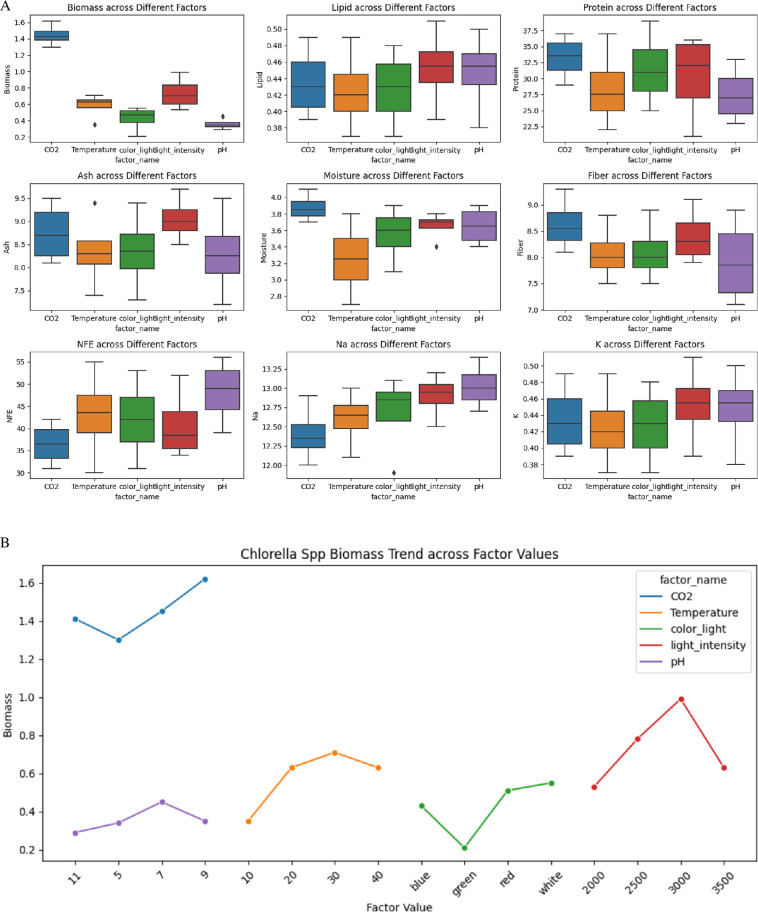
(A) Box plot shows the effect of various environmental factors on several biochemical and nutritional parameters of *Chlorella* species, including biomass, lipid, protein, ash, moisture, fibre, NFE, Na and K. (B) The plot illustrates *Chlorella* spp*.* biomass production trends across various environmental factors, including CO_2_ concentration, temperature, light colour, light intensity and pH.

Figure 3A illustrates the effects of various environmental factors on several biochemical parameters of *Chlorella* species, including biomass, lipid, protein, ash, moisture, fibre, NFE, Na and K. The box plots depict significant variations and distributions in these parameters under different conditions. Biomass exhibited the highest values under CO_2_ treatments, approximately 1.4 g l^−1^, and the lowest under pH adjustments, approximately 0.3−0.6 g l^−1^. Lipid content remained relatively consistent across treatments, with CO_2_ and pH treatments showing values approximately 0.42–0.50%. Protein content varied, peaking under CO_2_ treatments at 33–37%, while pH adjustments ranged from 22.5 to 35%. Ash content was highest under pH adjustments (8.5–9.5%) and CO_2_ treatments (8–9%). Moisture content was maximized under CO_2_ treatments (3.8–4.0%) and minimized under temperature treatments (3.2–3.6%). Fibre content showed the highest values under pH adjustments (8–9%), while other factors maintained consistent levels approximately 7–8.5%. NFE content remained relatively uniform, with light and temperature treatments showing higher percentages (40–50%) compared with CO_2_ and pH adjustments (35–45%). Sodium content varied significantly, with the highest levels under light conditions and pH adjustments (12.5−13.5 mg/100 g) and the lowest under CO_2_ treatments (12.0−12.5 mg/100 g). Potassium content remained consistent across treatments, with values approximately 0.42−0.48 mg/100 g. These findings are critical for optimizing growth conditions and enhancing the nutritional and industrial applications of *Chlorella* species.

For the optimization of *Chlorella* biomass (figure 3B) and nutrient composition, specific environmental factors have been identified as most effective. Maximum biomass production was achieved under a CO_2_ concentration of 9%, a temperature of 30°C, white light, a light intensity of 3000 lux and a pH of 7, yielding a biomass of 1.782 g l^−1^. For protein content, the optimal conditions included a CO_2_ concentration of 9%, a temperature of 30°C, red light, a light intensity of 3500 lux and a pH of 7, resulting in a maximum protein content of 42.9%. Lipid content was maximized with a CO_2_ concentration of 9%, a temperature of 30°C, red light, a light intensity of 3000 lux and a pH of 7, achieving a lipid content of 0.561 g l^−1^. Lastly, fibre content was highest under a CO_2_ concentration of 7%, a temperature of 30°C, white light, a light intensity of 3000 lux and a pH of 7, producing a fibre content of 10.23%.

### 3.5. The effect of environmental factors on biomass and other biochemical and nutritional compositions of *Botryococcus* spp.

**Figure 4.**
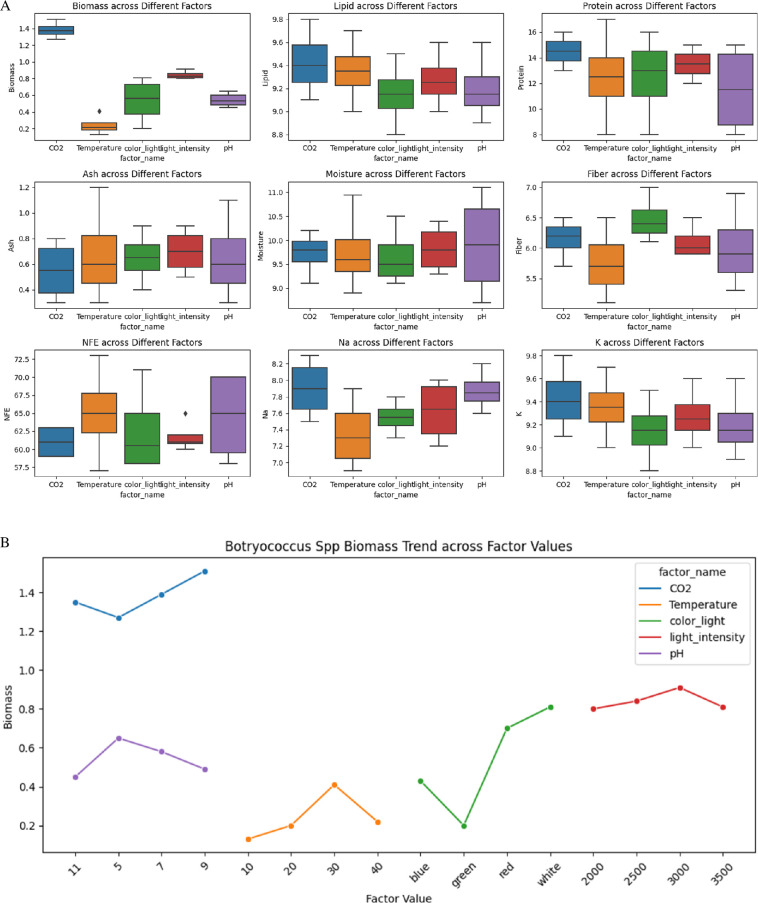
(A) Box plot shows the effect of various environmental factors on several biochemical and nutritional parameters of *Botryococcus* spp*.,* including biomass, lipid, protein, ash, moisture, fibre, NFE, Na and K. (B) The plot illustrates *Botryococcus* spp. biomass production trends across various environmental factors, including CO_2_ concentration, temperature, light colour, light intensity and pH.

The box plots in figure 4A depict significant variations and distributions in these parameters under different conditions. Biomass exhibited the highest values under CO_2_ treatments, approximately 1.4 g l^−1^, and the lowest under temperature adjustments, approximately 0.3 g l^−1^. Lipid content remained relatively consistent across treatments, with CO_2_ and pH treatments showing values approximately 9.4–9.2%. Protein content varied, peaking under CO_2_ treatments at 15–16%, while temperature adjustments ranged from 8 to 14%. Ash content was highest under temperature adjustments (approx. 0.8%) and lowest under CO_2_ treatments (approx. 0.6%).

Moisture content was maximized under pH adjustments (approx. 10.5%) and minimized under temperature treatments (approx. 10%). Fibre content showed the highest values under pH adjustments (6.5%), while other factors maintained consistent levels approximately 6%. NFE content remained relatively uniform, with light and temperature treatments showing higher percentages (approx. 66%) compared with CO_2_ and pH adjustments (approx. 63%).

Sodium content varied significantly, with the highest levels under CO_2_ conditions (approx. 7.8 mg l^−1^) and the lowest under light intensity treatments (approx. 7.4 mg l^−1^). Potassium content remained consistent across treatments, with values approximately 9.4−9.2 mg l^−1^. These findings are critical for optimizing growth conditions and enhancing the nutritional and industrial applications of *Botryococcus* spp.

Specific environmental conditions have been identified to optimize biomass (figure 4B) and nutrient content in *Botryococcus*. The maximum biomass yield of 1.66 g l^−1^ was achieved with a CO_2_ concentration of 9%, a temperature of 30°C, white light, a light intensity of 3000 lux and a pH of 5. Protein content was maximized at 18.7% under conditions of a CO_2_ concentration of 9%, a temperature of 30°C, red light, a light intensity of 3000 lux and a pH of 7. The highest lipid content, 10.78%, was observed with a CO_2_ concentration of 9%, a temperature of 30°C, red light, a light intensity of 3000 lux and a pH of 9. Fibre content reached its peak at 7.59% under a CO_2_ concentration of 7%, a temperature of 30°C, white light, a light intensity of 3000 lux and a pH of 7.

### 3.6. The effect of environmental factors on biomass and other biochemical and nutritional compositions of *Chlamydomonas* spp.

**Figure 5.**
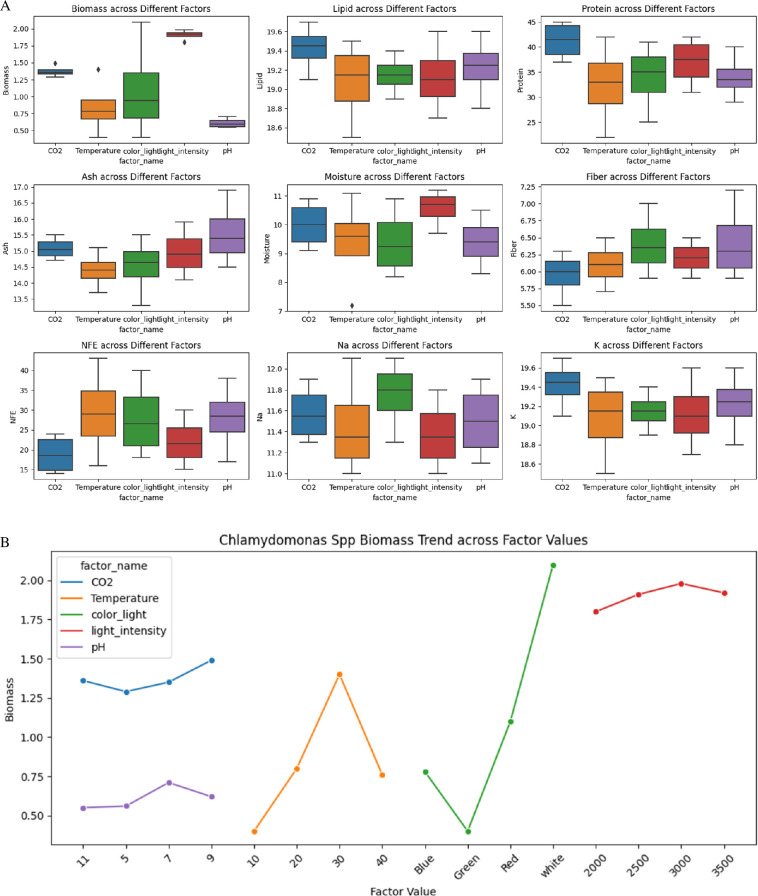
(A) Box plot shows the effect of various environmental factors on several biochemical and nutritional parameters of *Chlamydomonas* spp. including biomass, lipid, protein, ash, moisture, fibre, NFE, Na and K. (B) The plot illustrates *Chlamydomonas* spp. biomass production trends across various environmental factors, including CO_2_ concentration, temperature, light colour, light intensity and pH.

Biomass in figure 5 exhibited the highest values under CO_2_ treatments, with medians ranging from approximately 1.2 to 1.6 g l^−1^, and the lowest under pH adjustments, approximately 0.5 to 0.8 g l^−1^. Lipid content remained relatively consistent across treatments, with CO_2_ treatments showing values approximately 17.2–21.4%. Protein content varied significantly, peaking under CO_2_ treatments at 48.4–49.5%, while temperature adjustments ranged from 19.8 to 46.2%.

Ash content was highest under temperature adjustments (approx. 13.6–17.3%) and lowest under CO_2_ treatments (approx. 13.4%). Moisture content was maximized under pH adjustments (approx. 8.1–11.6%) and minimized under temperature treatments (approx. 6.5–12.2%). Fibre content showed the highest values under CO_2_ adjustments (6.9%), while other factors maintained consistent levels approximately 5.3–7.7%.

NFE content remained relatively uniform, with light intensity treatments showing higher percentages (approx. 21.6%) compared with CO_2_ and pH adjustments (approx. 12.6–24.2%). Sodium content varied significantly, with the highest levels under CO_2_ conditions (approx. 12.1–13.3 mg l^−1^) and the lowest under temperature treatments (approx. 10.0–12.6 mg l^−1^). Potassium content remained consistent across treatments, with values approximately 16.9–21.7 mg l^−1^.

The optimal environmental conditions for maximizing biomass, protein, lipid and fibre content in *Chlamydomonas* have been identified. The highest biomass yield of 1.639 g l^−1^ was observed at a CO_2_ concentration of 9%, a temperature of 30°C, white light, a light intensity of 3000 lux and a pH of 7. Protein content reached its peak at 49.5% under a CO_2_ concentration of 9%, a temperature of 30°C, red light, a light intensity of 3000 lux and a pH of 7. Lipid content was maximized at 21.67% with a CO_2_ concentration of 9%, a temperature of 30°C, red light, a light intensity of 3000 lux and a pH of 9. For fibre content, the highest value of 7.92% was achieved with a CO_2_ concentration of 9%, a temperature of 30°C, white light, a light intensity of 3000 lux and a pH of 9.

### 3.7. The effect of environmental factors on biomass and other biochemical and nutritional compositions of *Tetraselmis* spp.

**Figure 6.**
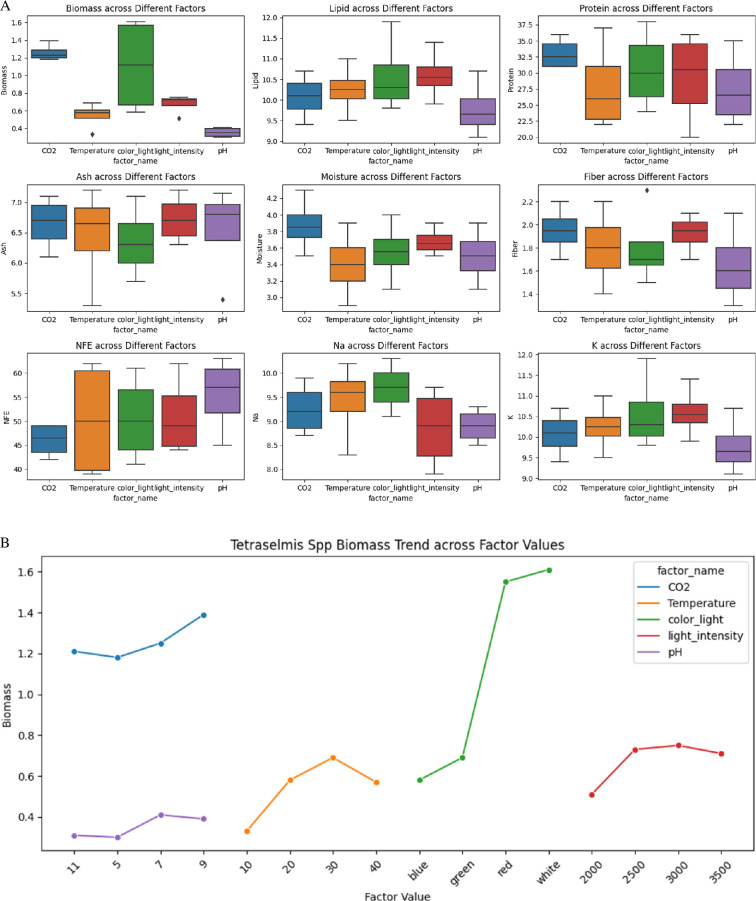
(A) Box plot shows the effect of various environmental factors on several biochemical and nutritional parameters of *Tetraselmis* spp. including biomass, lipid, protein, ash, moisture, fibre, NFE, Na and K. (B) The plot illustrates *Tetraselmis* spp. biomass production trends across various environmental factors, including CO_2_ concentration, temperature, light colour, light intensity, and pH.

The box plots reveal significant variations and distributions in these parameters under different conditions. Biomass exhibited the highest values under CO_2_ treatments, with medians ranging from approximately 1.1 to 1.5 g l^−1^, and the lowest under pH adjustments, approximately 0.27–0.45 g l^−1^ as depicted in figure 6A. Lipid content remained relatively consistent across treatments, with CO_2_ treatments showing values approximately 9.4–12.1%. Protein content varied significantly, peaking under CO_2_ treatments at 27.9–39.6%, while temperature adjustments ranged from 19.8 to 37.4%. Ash content was highest under CO_2_ treatments (approx. 5.85–7.15%) and lowest under pH adjustments (approx. 6.03%). Moisture content was maximized under pH adjustments (approx. 2.79–7.86%) and minimized under temperature treatments (approx. 2.61–4.73%). Fibre content showed the highest values under CO_2_ adjustments (4.73%), while other factors maintained consistent levels approximately 1.3–3.85%. NFE content remained relatively uniform, with light intensity treatments showing higher percentages (approx. 40.5–49.5%) compared with CO_2_ and pH adjustments (approx. 36–69.3%). Sodium content varied significantly, with the highest levels under CO_2_ conditions (approx. 7.83–10.89 mg l^−1^) and the lowest under temperature treatments (approx. 7.47–10.67 mg l^−1^). Potassium content remained consistent across treatments, with values approximately 8.82–11.77 mg l^−1^. These findings are critical for optimizing growth conditions and enhancing the nutritional and industrial applications of *Tetraselmis* spp*.*

The optimal environmental conditions for maximizing biomass (figure 6B) and nutrient composition in *Tetraselmis* have been determined. The highest biomass yield of 1.52 g l^−1^ was obtained with a CO_2_ concentration of 9%, a temperature of 30°C, white light, a light intensity of 3000 lux and a pH of 7. Protein content was maximized at 41.8% under conditions of a CO_2_ concentration of 9%, a temperature of 30°C, red light, a light intensity of 3500 lux and a pH of 7. The maximum lipid content of 13.09% was achieved with a CO_2_ concentration of 9%, a temperature of 30°C, red light, a light intensity of 3000 lux and a pH of 7. Fibre content reached its peak at 2.53% under conditions of a CO_2_ concentration of 9%, a temperature of 30°C, white light, a light intensity of 3000 lux and a pH of 7. These findings highlight the significant impact of environmental factors on *Tetraselmis*’ biomass and nutrient profile, underscoring its potential for targeted biotechnological applications.

### 3.8. The effect of environmental factors on biomass and other biochemical and nutritional compositions of *Closterium* spp.

**Figure 7.**
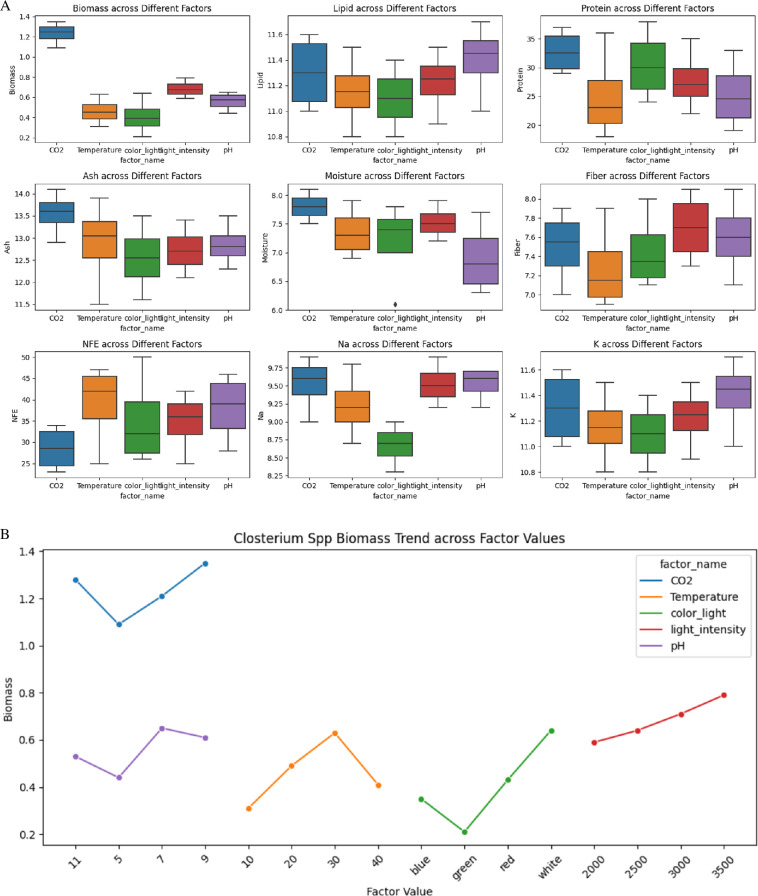
(A) Box plot shows the effect of various environmental factors on several biochemical and nutritional parameters of *Closterium* spp. including biomass, lipid, protein, ash, moisture, fibre, NFE, Na and K. (B) The plot illustrates *Closterium* spp. biomass production trends across various environmental factors, including CO_2_ concentration, temperature, light colour, light intensity and pH.

Biomass exhibited the highest values under CO_2_ treatments, with medians ranging from approximately 1.2 to 1.4 g l^−1^, and the lowest under pH adjustments, approximately 0.5 to 0.8 g l^−1^ as illustrated in figure 7A. Lipid content remained relatively consistent across treatments, with CO_2_ treatments showing values approximately 11.2–11.6%. Protein content varied significantly, peaking under CO_2_ treatments at 35.1–49.5%, while temperature adjustments ranged from 19.8 to 46.2%.

Ash content was highest under temperature adjustments (approx. 12.6–13.6%) and lowest under CO_2_ treatments (approx. 13.4%). Moisture content was maximized under pH adjustments (approx. 6.9–8.2%) and minimized under temperature treatments (approx. 6.2–7.1%). Fibre content showed the highest values under CO_2_ adjustments (7.7%), while other factors maintained consistent levels approximately 7.2–8.2%.

NFE content remained relatively uniform, with light intensity treatments showing higher percentages (approx. 35.1–49.5%) compared with CO_2_ and pH adjustments (approx. 12.6–34.2%). Sodium content varied significantly, with the highest levels under CO_2_ conditions (approx. 9.2–10.7 mg l^−1^) and the lowest under temperature treatments (approx. 8.2–10.5 mg l^−1^). Potassium content remained consistent across treatments, with values approximately 10.1–11.2 mg l^−1^.

The conditions for maximizing biomass (figure 7B) and nutrient composition in *Closterium* have been identified. The highest biomass yield of 1.485 g l^−1^ was achieved with a CO_2_ concentration of 9%, a temperature of 30°C, white light, a light intensity of 3500 lux and a pH of 7. Protein content reached its peak at 41.8% under conditions of a CO_2_ concentration of 9%, a temperature of 30°C, red light, a light intensity of 3000 lux and a pH of 7. The maximum lipid content of 12.87% was observed with a CO_2_ concentration of 9%, a temperature of 30°C, red light, a light intensity of 3000 lux and a pH of 9. Fibre content was highest at 8.91% under a CO_2_ concentration of 9%, a temperature of 30°C, white light, a light intensity of 3000 lux and a pH of 9. These findings demonstrate that *Closterium*’s biomass and nutrient profile can be significantly influenced by specific environmental parameters, highlighting its potential for biotechnological applications.

### 3.9. The average effect of environmental factors on biomass and other biochemical and nutritional compositions of all species included in the study

**Figure 8.**
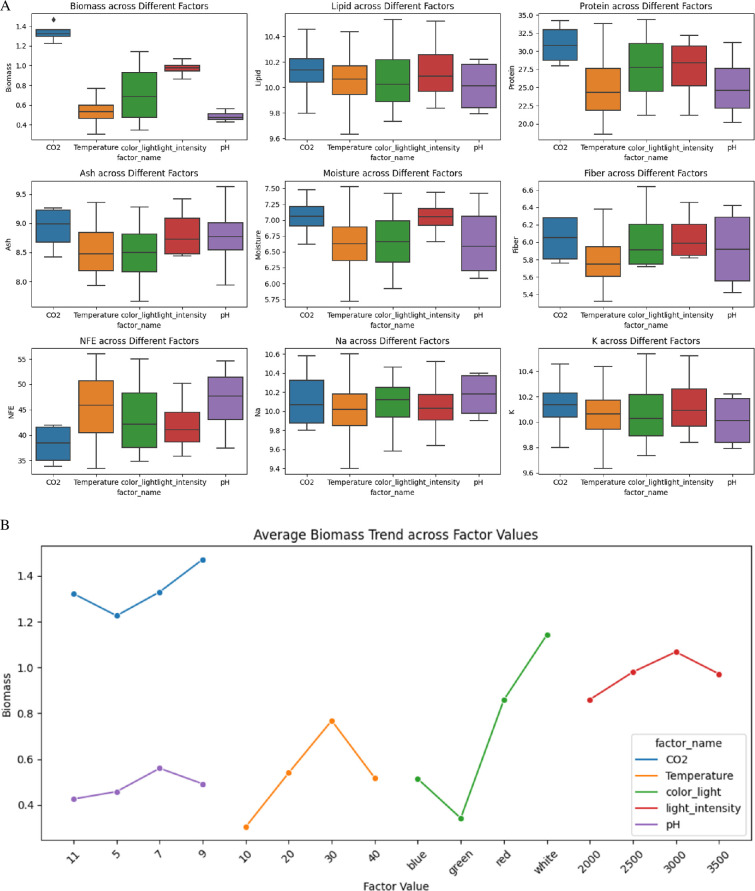
(A) Box plot shows the average effect of various environmental factors on several biochemical and nutritional parameters of all algae species including biomass, lipid, protein, ash, moisture, fibre, NFE, Na and K. (B) The plot illustrates average biomass production trends across various environmental factors, including CO_2_ concentration, temperature, light colour, light intensity and pH.

The data in figure 8A reveal notable variations in these parameters under different conditions. Biomass was maximized under CO_2_ treatments, with median values ranging from 1.1 to 1.4 g l^−1^, and minimized under pH adjustments, showing values between 0.4 and 0.6 g l^−1^. Lipid content was highest under CO_2_ and pH treatments, ranging from 10.8 to 11.6%, while temperature adjustments resulted in the lowest lipid content, between 9.0 and 10.4%. Protein content peaked under CO_2_ conditions, with values from 25.2 to 37.6%, and was lowest under pH adjustments, ranging from 18.2 to 34.3%.

Ash content was elevated under CO_2_ and temperature treatments, approximately 8.0–9.5%, and lowest under pH adjustments, approximately 7.0–8.5%. Moisture content was maximized with CO_2_ treatments, between 7.0 and 7.5%, and minimized under pH adjustments, approximately 5.8–6.2%. Fibre content was highest under CO_2_ and temperature conditions, approximately 6.2–6.8%, and lowest under pH adjustments, from 5.4 to 5.8%. NFE content was maximized under pH adjustments, ranging from 50.4 to 60.1%, and minimized under CO_2_ treatments, between 30.4 and 38.0%. Sodium content was highest with CO_2_ treatments, from 9.2 to 11.4 mg l^−1^, and lowest under temperature adjustments, from 8.5 to 10.0 mg l^−1^.

Potassium content peaked under CO_2_ and pH conditions, approximately 9.5–11.1 mg l^−1^, and was lowest under temperature adjustments, between 8.0 and 10.5 mg l^−1^. These results underscore the importance of CO_2_ treatments for optimizing growth conditions and enhancing the nutritional and industrial value of these algae species, while pH adjustments generally minimized most parameters, except for NFE content. Temperature adjustments typically resulted in lower lipid and sodium content while maintaining moderate values for other parameters.

The analysis of average values across all species for optimizing biomass (figure 8B) and nutrient composition has revealed specific environmental conditions. Maximum biomass production, with an average yield of 1.61 g l^−1^, was achieved under a CO_2_ concentration of 9%, a temperature of 30°C, white light, a light intensity of 3000 lux and a pH of 7. For protein content, the optimal conditions included a CO_2_ concentration of 9%, a temperature of 30°C, red light, a light intensity of 3000 lux and a pH of 7, resulting in an average protein content of 37.84%. The highest average lipid content of 11.58% was observed with a CO_2_ concentration of 9%, a temperature of 30°C, red light, a light intensity of 3000 lux and a pH of 7. Fibre content was maximized at an average of 7.30% under conditions of a CO_2_ concentration of 7%, a temperature of 30°C, white light, a light intensity of 3000 lux and a pH of 7. These findings illustrate the significant influence of environmental parameters on the biomass and nutrient profile across different species, highlighting the potential for optimizing conditions in biotechnological applications.

## 4. Discussion

The optimization of biomass production and nutrient composition in various algal genera (*Chlorella*, *Botryococcus*, *Chlamydomonas*, *Tetraselmis* and *Closterium*) is significantly influenced by environmental factors such as pH, temperature, coloured light, light intensity and CO_2_ concentration. *Chlamydomonas* spp. demonstrated the highest biomass yield of 2.1 g l^−1^ under white light, with optimal growth conditions at pH 7, a temperature of 30°C, a light intensity of 3000 lux and a CO_2_ concentration of 9%. These conditions align with studies by Visviki *et al*. [43], who reported maximum algal growth at neutral pH, and by Daliry [44], who found that temperatures between 25 and 30°C are optimal for *Chlorella vulgaris*. High light intensities and specific wavelengths also play crucial roles in biomass accumulation, as reported by Fu *et al*. [45] and Hegemann [46], who highlighted the influence of light quality on photosynthetic efficiency and cell division processes.

For lipid content, *Chlamydomonas* spp. achieved a maximum of 24.5% under conditions of pH 9, a temperature of 30°C, red light and a light intensity of 3000 lux. High CO_2_ concentrations also enhanced lipid production, consistent with findings by Juneja *et al*. [47], who noted increased lipid accumulation at neutral pH, and Moheimani [48], who optimized lipid productivity in *Tetraselmis suecica* and *Chlorella* spp. through pH adjustments. The positive impact of red light on lipid biosynthesis was also supported by Khotimchenko & Yakovleva [49].

Protein content in *Chlamydomonas* spp. was maximized at 45% under pH 7, a temperature of 30°C, red light, a light intensity of 3000 lux and 9% CO_2_ concentration. These results align with studies by Carvalho *et al*. [50], who demonstrated that neutral pH and specific light wavelengths enhance protein synthesis, and Konopka & Brock [51], who showed that deviations from optimal temperatures inhibit protein synthesis by affecting enzymatic activities.

The highest fibre content (9.3%) in *Chlorella* spp. was observed under pH 7, a temperature of 30°C, white light, a light intensity of 3000 lux and 7% CO_2_ concentration. This is consistent with findings by Ogbonda *et al*. [52], who reported that optimal pH conditions enhance cell wall rigidity and fibre production. The role of light intensity and quality in influencing fibre content is also highlighted by Blair *et al*.’s [53] work.

Environmental factors such as pH, temperature, light quality, light intensity and CO_2_ concentration significantly impact ash content, moisture content and mineral composition, including sodium and potassium levels, across various algal species. High ash content, which indicates higher mineral content, can be detrimental if it exceeds recommended levels for nutraceutical applications [54,55]. Other studies [56,57] demonstrated the positive relationship between these environmental factors and mineral uptake in algae. These findings underscore the importance of optimizing environmental conditions to enhance the biomass and nutrient profile of algae, with significant implications for biotechnological applications and algal cultivation strategies.

**Figure 9.**
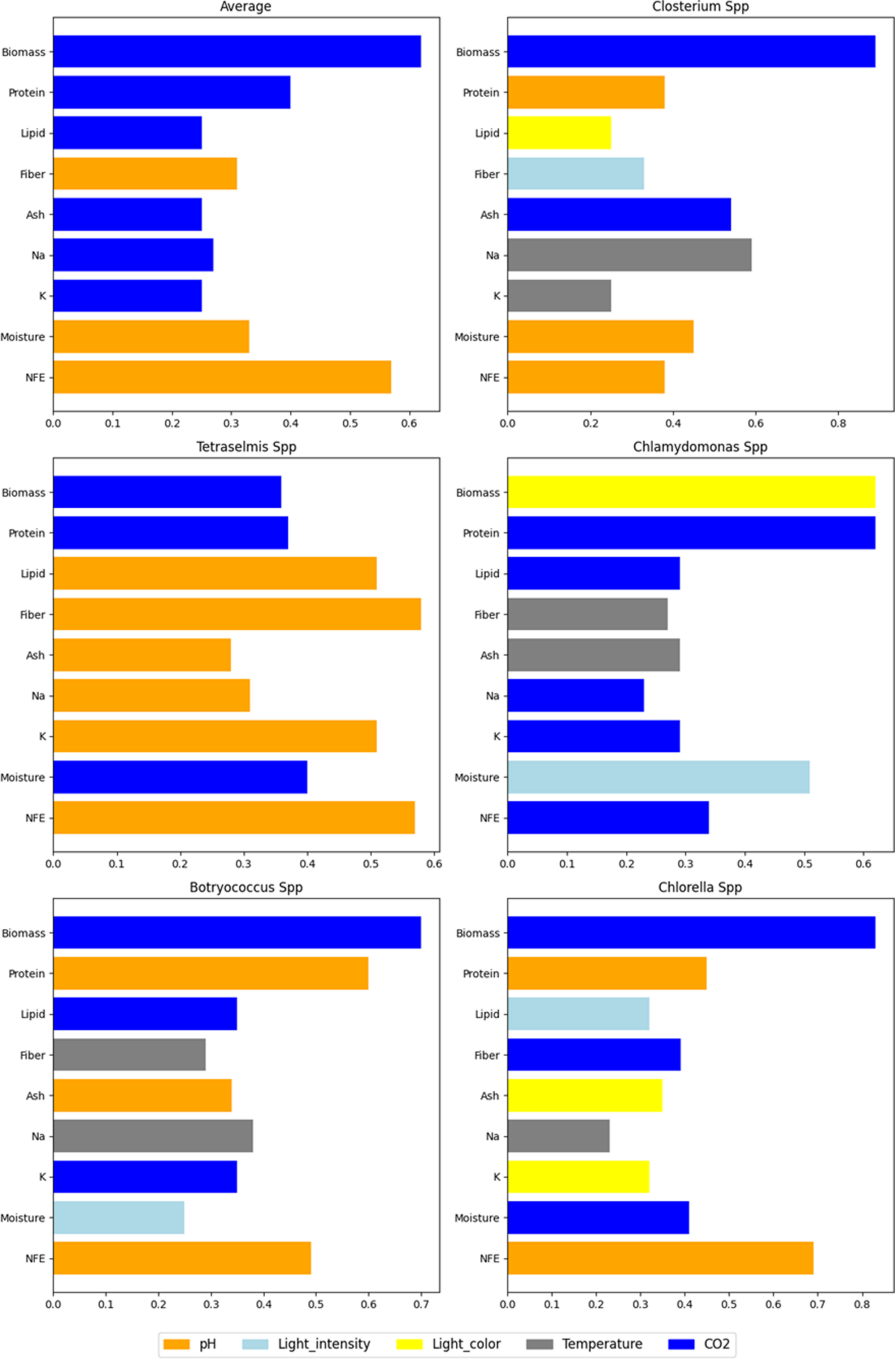
The figure illustrates the environmental factor with the highest effect on each biochemical and nutritional component (biomass, protein, lipid, fibre, ash, Na, K, moisture, NFE) for six categories: the average across all genera and, for each genus, separately. The colours represent different factors: pH (orange), light Intensity (light blue), light colour (yellow), temperature (grey) and CO_2_ (blue). The length of the bars indicates the magnitude of the factor’s influence, showing that CO_2_ generally has the most significant impact on biomass, while pH significantly affects NFE across species.

In *Closterium* spp., CO_2_ emerged as the most critical factor for biomass (0.89) and ash (0.54), while pH was the most significant predictor for protein content (0.38). For *Tetraselmis* spp., CO_2_ and pH were highly influential for biomass (0.35) and protein content (0.32), respectively. In *Chlamydomonas* spp., light intensity was the most crucial factor for biomass (0.62), while CO_2_ significantly influenced protein content (0.62). *Botryococcus* spp. showed CO_2_ as the most important factor for biomass (0.69) and lipid content (0.34), with pH being critical for protein content (0.60). *Chlorella* spp. had CO_2_ as the key factor for biomass (0.82), while pH was highly significant for protein content (0.44). Across different algal species, CO_2_ consistently influenced biomass, protein and lipid contents, while pH was frequently significant for protein and NFE. Refer to figure 9 to see the environmental factor that has the highest effect on biomass and other biochemical and nutritional components in each algae species.

Light intensity was crucial for lipid and ash contents, and temperature showed varying levels of influence across different variables, being significant for fibre and ash in many cases. Light colour had a notable impact on sodium and potassium. Key numerical findings include CO_2_’s influence on biomass (0.89 in *Closterium*, 0.82 in *Chlorella*), pH’s significance for protein (0.38 in *Closterium*, 0.44 in *Chlorella*) and light intensity’s importance for lipid content (0.32 in *Chlorella*, 0.20 in *Tetraselmis*). These results, illustrated in electronic supplementary material, part 3, suggest that optimizing different factors can enhance specific aspects of algae growth and composition. For instance, focusing on CO_2_ levels can significantly improve biomass and protein contents, while adjusting pH can enhance protein and NFE content.

The correlation analysis performed in this study provides further insights into the relationships between environmental factors and biochemical properties, enhancing the robustness of our findings. The analysis revealed minimal correlations among environmental factors, such as pH, temperature, CO₂ concentration, light intensity and light colour, with coefficients generally close to zero (e.g. CO₂ and temperature: −0.25). This indicates that the experimental design effectively controlled for mutual influence among these factors, allowing for independent evaluation of their effects on biochemical properties, as illustrated in electronic supplementary material, figure S1.

Furthermore, strong correlations were observed between certain biochemical properties, such as lipid and ash (correlation coefficient: 0.89), which probably reflect inherent metabolic or compositional relationships within the algal species. These correlations are consistent with known biochemical pathways and do not imply confounding effects. Notably, CO₂ showed a strong positive correlation with biomass (0.73) and a moderate correlation with protein (0.34), confirming its critical role in enhancing algal growth and nutrient accumulation. These findings align with the experimental results and underscore the importance of optimizing CO₂ levels to achieve higher yields in biomass and nutrient content.

### 4.1. Average results of all species

The aggregated results from all algal species demonstrated that the factors significantly influenced the dependent variables, as confirmed by the MANOVA tests. For biomass, CO_2_ was the most important factor with a feature importance score of 0.61, followed by light intensity (0.22). For protein content, CO_2_ (0.40) and pH (0.37) were highly influential. Lipid content was most affected by CO_2_ (0.24) and pH (0.21), while fibre content was primarily influenced by pH (0.30) and temperature (0.21). Ash content showed the highest dependence on CO_2_ (0.25) and light intensity (0.23). For Na, light colour (0.21) and CO_2_ (0.26) were the most critical factors. K content was significantly influenced by CO_2_ (0.24) and light intensity (0.17). Moisture content was primarily affected by pH (0.32) and light intensity (0.21), while NFE was most influenced by pH (0.56) and CO_2_ (0.19). These results underscore the critical role of CO_2_ and pH across various growth parameters, suggesting that optimizing these factors could significantly enhance algal growth and composition. Focusing on CO_2_ levels can improve biomass and protein content, while adjusting pH can enhance protein, fibre and NFE contents.

These average effect analyses serve as the foundation for the ML models employed in this study. By identifying the environmental factors with the greatest impact on multiple microalgal growth and biochemical composition, the models prioritize these variables for optimization. The ML approach then integrates these insights to predict optimal conditions for maximizing desirable outcomes, such as biomass and protein content, across all species. This process enables a generalized optimization framework that accounts for inter-species variability while focusing on the most influential factors.

The MANOVA analysis plays a critical role in the optimization process by statistically validating the significance of environmental factors on multiple biochemical properties simultaneously. This multivariate approach ensures that the relationships between environmental factors and algal growth are robust and not confounded by mutual influences among the biochemical parameters. The insights gained from MANOVA inform the ML models by identifying the most influential factors, which are then prioritized for predictive analysis and optimization. By integrating MANOVA results with ML, this study achieves a more comprehensive framework for optimizing algal growth and biochemical composition.

### 4.2. Advancements in algal biotechnology through machine learning applications

ML has emerged as a transformative tool in algal research, offering advanced methods to analyse complex datasets and optimize cultivation processes [58]. By leveraging ML algorithms, researchers can identify critical environmental variables that significantly influence algal growth and biochemical composition [59]. This capability enables the development of predictive models that accurately forecast algal responses to varying conditions, facilitating the fine-tuning of growth parameters to enhance biomass yield and the production of valuable metabolites [60,61]. Consequently, ML-driven approaches contribute to more efficient and sustainable algal cultivation practices, supporting advancements in biotechnology, biofuel, medicines and related fields [62–64].

ML has been effectively applied in algal research to optimize cultivation processes and enhance the production of valuable compounds. For instance, researchers utilized a genetic algorithm to refine the culture medium for *Nannochloropsis gaditana*, resulting in a 23% increase in EPA productivity, achieving 17.8 mg l^−1^ d^−1^. This optimization also led to significant reductions in nutrient requirements, including decreases of 74% for phosphorus, 69% for molybdenum, 66% for manganese and 46% for thiamine [65]. Additionally, a comprehensive review highlighted the application of ML techniques across various stages of algal biofuel production, such as strain selection, cultivation optimization and metabolic engineering. The study emphasized that ML models can predict algal growth and lipid accumulation under different environmental conditions, thereby facilitating the development of efficient biofuel production processes [66]. Furthermore, the Fraunhofer Institute for Interfacial Engineering and Biotechnology (IGB) applied support vector machines to predict the growth behaviour of the microalga *Phaeodactylum tricornutum* in outdoor cultivation, enabling the development of robust, predictive control systems for large-scale algal cultivation [67]. These examples underscore the versatility and effectiveness of ML models in enhancing algal biomass production and metabolite extraction, highlighting their potential to drive advancements in sustainable biotechnology.

The integration of ML into algal research holds significant promise for advancing sustainable biotechnological applications [68]. By optimizing cultivation conditions through ML algorithms, researchers can enhance the efficiency of biomass production and the extraction of bioactive compounds. This optimization leads to improved resource utilization and reduced production costs, which are crucial for the development of eco-friendly biofuels and pharmaceuticals [69,70]. Moreover, the predictive capabilities of ML facilitate the design of cultivation strategies that maximize yield while minimizing environmental impact, aligning with global sustainability goals. As the field progresses, the continued application of ML is expected to drive innovations in algal biotechnology, contributing to a more sustainable and resilient bioeconomy [71].

## 5. Conclusion

This study underscores the critical role of environmental factors in optimizing the biomass yield and biochemical composition of microalgae, presenting a comprehensive framework for sustainable biofuel production and biotechnological applications. Through an integrated approach of experimental optimization and ML, we systematically investigated the effects of pH, temperature, light intensity, light quality and CO₂ concentration on five algal genera (*Chlorella* spp.*, Botryococcus* spp.*, Chlamydomonas* spp*., Tetraselmis* spp*.,* and *Closterium* spp*.*).

Among the genera, *Chlamydomonas* spp. demonstrated the highest productivity, achieving biomass of 2.1 g l^−1^ and protein content of 49.5% under optimal conditions: pH 7, 30°C, red light, 3000 lux and 9% CO₂ concentration. Lipid production peaked at 24.5% under slightly alkaline conditions (pH 9) with red light exposure, while fibre and NFE contents were significantly enhanced through fine-tuned pH and CO₂ adjustments. These results highlight the differential sensitivity of algal species to specific environmental parameters.

Advanced ML models, particularly RF regressors, were instrumental in identifying CO₂ concentration and pH as the most critical drivers of growth and nutrient optimization. RF models were rigorously evaluated using a novel composite metric (*W*_new), which integrated *R*², MAE, RMSE and m.s.e. to ensure robust predictions. The RF model achieved an *R*² of 0.686 (training) and 0.534 (validation), outperforming other models such as KNeighbors, SVR and Gradient Boosting. Hyperparameter tuning using GridSearchCV further optimized the model, with n_estimators set to 100, max_depth to 10, min_samples_split to 5 and min_samples_leaf to 5, providing a balance between predictive accuracy and computational efficiency. Feature importance analysis revealed CO₂ and pH as dominant factors influencing biomass and biochemical traits, supported by multivariate statistical validation using MANOVA.

This study establishes a scalable methodology for microalgal cultivation, bridging computational insights with experimental validation. The integration of ML not only refined predictive accuracy but also provided actionable insights for optimizing environmental parameters. The findings pave the way for future research on the synergistic effects of environmental and genetic factors, enhanced photobioreactor designs and resource-efficient cultivation strategies. This transformative approach lays a foundation for advancing algal biotechnology and achieving a sustainable bioeconomy.

## Data Availability

All data are included in the manuscript; for further information, the correspondence author can be contacted. Supplementary material is available online [72].
